# Central nervous system efficacy of furmonertinib (AST2818) in patients with *EGFR* T790M mutated non-small cell lung cancer: a pooled analysis from two phase 2 studies

**DOI:** 10.1186/s12916-023-02865-z

**Published:** 2023-04-28

**Authors:** Xingsheng Hu, Shucai Zhang, Zhiyong Ma, Jifeng Feng, Lin Wu, Dongqing Lv, Jianying Zhou, Xiaodong Zhang, Li Liu, Qitao Yu, Wangjun Liao, Yiping Zhang, Xiang Wang, Ying Cheng, Hongrui Niu, Ziping Wang, Dong Wang, Cheng Huang, Chunling Liu, Hui Zhao, Jian Feng, Jingzhang Li, Kejing Ying, Nong Yang, Shukui Qin, Jie Hu, Fei Liu, Yong Jiang, Nan Ge, Yuankai Shi

**Affiliations:** 1 Department of Medical Oncology, National Cancer Center/National Clinical Research Center for Cancer/Cancer Hospital, Chinese Academy of Medical Sciences & Peking Union Medical College, Beijing Key Laboratory of Clinical Study On Anticancer Molecular Targeted Drugs, Beijing, 100021 China; 2 Department of Oncology, Beijing Chest Hospital, Capital Medical University, Beijing Tuberculosis and Thoracic Oncology Institute, Beijing, China; 3 Department of Medical Oncology, Affiliated Cancer Hospital of Zhengzhou University/Henan Cancer Hospital, Zhengzhou, China; 4 Department of Oncology, Jiangsu Cancer Hospital & Jiangsu Institute of Cancer Research, Nanjing Medical University Affiliated Cancer Hospital, Nanjing, China; 5 Thoracic Medicine Department II, Hunan Cancer Hospital, The Affiliated Cancer Hospital of Xiangya School of Medicine, Central South University, Changsha, China; 6 Department of Respiratory Medicine, Taizhou Hospital of Zhejiang Province, Taizhou, China; 7 Department of Respiratory Medicine, The First Affiliated Hospital, Zhejiang University School of Medicine, Hangzhou, China; 8 Department of Medical Oncology, Nantong Cancer Hospital, Nantong, China; 9 Department of Thoracic Oncology, Cancer Center, Union Hospital, Tongji Medical College, Huazhong University of Science and Technology, Wuhan, China; 10 Department of Respiratory Oncology, Guangxi Medical University Affiliated Tumor Hospital, Nanning, China; 11 Department of Oncology, Nanfang Hospital, Southern Medical University, Guangzhou, China; 12 Department of Medical Oncology, Zhejiang Cancer Hospital, Hangzhou, China; 13 Department of Medical Oncology, Xuzhou Central Hospital, Xuzhou, China; 14 Department of Oncology, Jilin Cancer Hospital, Changchun, China; 15 Department of Oncology, The First Affiliated Hospital of Xinxiang Medical University, Xinxiang, China; 16 Department of Thoracic Medical Oncology, Peking University Cancer Hospital and Institute, Beijing, China; 17 Department of Oncology, Army Medical Centre of People’s Liberation Army, Chongqing, China; 18 Department of Oncology, Fujian Cancer Hospital, Fuzhou, China; 19 Department of Pulmonary Medicine, Cancer Hospital of Xinjiang Medical University, Urumqi, China; 20 Department of Respiratory and Critical Care Medicine, The Second Hospital of Anhui University, Hefei, China; 21 Department of Respiratory Medicine, Affiliated Hospital of Nantong University, Nantong, China; 22 Department of Oncology, Liuzhou People’s Hospital, Liuzhou, China; 23 Department of Respiratory Medicine, Sir Run Shaw Hospital, Zhejiang University School of Medicine, Zhejiang University, Hangzhou, China; 24 Department of Medical Oncology, Lung Cancer and Gastrointestinal Unit, Hunan Cancer Hospital, the Affiliated Cancer Hospital of Xiangya School of Medicine, Central South University, Changsha, China; 25 The People’s Liberation Army Cancer Center, Jinling Hospital, Nanjing, China; 26 Shanghai Allist Pharmaceutical Technology Co., Ltd, , Shanghai, China

**Keywords:** NSCLC, *EGFR*, Furmonertinib, AST2818, CNS

## Abstract

**Background:**

Furmonertinib (AST2818) is a brain penetrant pan-epidermal growth factor receptor (EGFR) tyrosine kinase inhibitor (TKI) targeting both *EGFR* sensitizing mutations and T790M mutation. We report the pooled central nervous system (CNS) efficacy data of furmonertinib in patients with *EGFR* T790M mutated non-small cell lung cancer (NSCLC) from two phase 2 studies.

**Methods:**

This was a pooled, post-hoc analysis of two phase 2 studies (NCT03127449 [phase 2a study of furmonertinib], NCT03452592 [phase 2b study of furmonertinib]). In the phase 2a study, patients received furmonertinib 40 mg, 80 mg, 160 mg, or 240 mg orally once daily. In the phase 2b study, all patients received furmonertinib 80 mg orally once daily. CNS efficacy of furmonertinib was analyzed in patients with baseline CNS lesions by an independent review center per Response Evaluation Criteria in Solid Tumors version 1.1.

**Results:**

A total of 132 patients with baseline CNS metastases were included in this analysis. In 52 patients with measurable CNS lesions, CNS objective response rates were zero (0/1), 65% (22/34), 85% (11/13), and 25% (1/4), and CNS disease control rates were zero (0/1), 97% (33/34), 100% (13/13), and 100% (4/4) in the 40 mg, 80 mg, 160 mg, and 240 mg orally once daily group, respectively. In patients with measurable or non-measurable CNS lesions, median CNS progression-free survival was 2.8 months (95% confidence interval [CI] 1.4–8.3), 11.6 months (95% CI 8.3–13.8), 19.3 months (95% CI 5.5-not available [NA]), and not reached (95% CI 2.8 months-NA) in the 40 mg, 80 mg, 160 mg, and 240 mg orally once daily group, respectively.

**Conclusions:**

Furmonertinib showed promising CNS efficacy in doses of 80 mg orally once daily or higher in patients with *EGFR* T790M mutated NSCLC.

**Trial registration:**

Both studies were registered on ClinicalTrial.gov. The phase 2a study was registered with NCT03127449 on April 25, 2017; The phase 2b study was registered with NCT03452592 on March 2, 2018.

**Supplementary Information:**

The online version contains supplementary material available at 10.1186/s12916-023-02865-z.

## Background

Epidermal growth factor receptor (*EGFR*) sensitizing mutations were the most frequent driven alterations detected in non-small cell lung cancer (NSCLC), especially in Asian patients with lung adenocarcinoma [1, 2]. Treatment with first- and second-generation EGFR tyrosine kinase inhibitors (TKIs) had prolonged the median progression-free survival (PFS) to 9.2–14.7 months in *EGFR* mutated advanced NSCLC patients [3–7], which was superior to chemotherapy and changed the treatment landscape of NSCLC dramatically. Nevertheless, resistance would inevitably occur and the most common reason for first- and second-generation EGFR TKI treatment failure was a secondary *EGFR* T790M mutation.

Several covalent pyrimidine-based third-generation EGFR TKIs such as osimertinib and rociletinib were developed to act against *EGFR* T790M mutation [8, 9]. Osimertinib was the first approved third-generation EGFR TKI for the first-line treatment of patients with metastatic NSCLC whose tumors have *EGFR* 19Del or L858R mutations and for the treatment of patients with metastatic *EGFR* T790M mutation-positive NSCLC whose disease has progressed on or after EGFR TKI therapy by the US Food and Drug Administration (FDA) [10–13].

Brain metastases (BM) were commonly reported in patients with NSCLC and were associated with poor survival outcomes [14]. Compared with unselected patients, NSCLC patients with *EGFR* sensitizing mutations were found to have a higher incidence of BM [15]. Therefore, it is crucial to develop EGFR TKIs with central nervous system (CNS) activity and to evaluate the CNS efficacy in clinical studies irrespective of treatment lines.

Furmonertinib (AST2818) is a brain penetrant pan-EGFR TKI developed by Shanghai Allist Pharmaceuticals Co., Ltd, Shanghai, China. It has a trifluoroethoxypyridine-based molecule structure and can irreversibly inhibit *EGFR* T790M mutation with high selectivity [16]. In preclinical studies, the concentration of furmonertinib and its main active metabolite in the brain was higher than that in the plasma, indicating that furmonertinib had the potential to treat CNS metastases [16]. In a phase 2b study, the CNS objective response rate (ORR) of furmonertinib 80 mg orally once daily in *EGFR* T790M mutated NSCLC patients was 66% and the median CNS-PFS was 11.6 months [17]. In the phase 3 FURLONG study, furmonertinib was associated with higher PFS and CNS-PFS compared with gefitinib in *EGFR* sensitizing mutation positive, untreated, CNS metastatic patients [18, 19]. These data had demonstrated the CNS efficacy of furmonertinib 80 mg orally once daily, but the evidence of other doses for CNS metastases was lacking. Here we report the CNS efficacy of different doses of furmonertinib in *EGFR* T790M mutated, CNS metastatic NSCLC patients, along with the exploratory genetic analysis. FURLONG study was excluded in this analysis because it was a first-line study (patients with common *EGFR* sensitizing mutations without T790M mutation), whereas the pooled analysis of two phase 2 studies (NCT03127449 [phase 2a study of furmonertinib], NCT03452592 [phase 2b study of furmonertinib]) was focused on furmonertinib in pre-treated patients with T790M mutation.

## Methods

### Study design and procedures

This was a pooled, post-hoc analysis of two phase 2 studies (NCT03127449 [phase 2a study of furmonertinib], NCT03452592 [phase 2b study of furmonertinib]). Both studies were open-label, single-arm, multi-center study, which conducted in 14 and 46 hospitals in the People’s Republic of China, respectively. Eligible patients received furmonertinib 40 mg, 80 mg, 160 mg, or 240 mg orally once daily in the phase 2a study and received furmonertinib 80 mg orally once daily in the phase 2b study, both until disease progression or any other cessation criterion was met.

Blood samples were collected at baseline and six weeks after treatment for exploratory genetic analysis. The genetic analysis was done with next-generation sequencing (NGS, Tongshu Biotech, Shanghai, China), a target panel of 556 genes with mean sequencing depths of more than 7000 times, detailed information could be found in a previous publication [17].

### Patients

For detailed information on patient inclusion and exclusion criteria, please refer to two previous publications by Shi Y et al. [16, 17]. Briefly, eligible patients in both studies were aged at least 18 years old, with locally advanced or metastatic NSCLC diagnosed histologically or cytologically, had measurable diseases as defined by the Response Evaluation Criteria in Solid Tumors (RECIST) version 1.1, had radiologically documented disease progression on first- or second-generation EGFR TKIs, with *EGFR* T790M mutation confirmed using tumor tissue by the central lab, with Eastern Cooperative Oncology Group (ECOG) performance status (PS) of 0–2. Patients with asymptomatic, stable CNS metastases which did not require steroids for four weeks or more before the first dose of furmonertinib were allowed for enrolment.

### Assessments

Tumor assessments were done every six weeks for the first 48 weeks, and then every 12 weeks until disease progression. Baseline brain scans (computerized tomography or magnetic resonance imaging) were mandated for all patients at study entry. Subsequent brain imaging was required with the same method used at baseline when clinically indicated and in patients with confirmed CNS metastases.

The patients with baseline CNS metastases according to an independent review center (IRC) constituted this analysis. Patients were grouped based on the doses of furmonertinib. The endpoints in this report included CNS-ORR, CNS disease control rate (DCR), CNS-PFS, and CNS duration of response (DoR) of each group. Patients with measurable or non-measurable CNS lesions were defined as CNS full analysis set (cFAS). Patients with measurable CNS lesions were defined as CNS evaluable-for-response set (cEFR). All the CNS measurements were assessed by an IRC according to the RECIST version 1.1.

CNS-ORR was defined as the proportion of patients who achieved complete response (CR) or partial response (PR) in CNS lesions with subsequent confirmation. CNS-DCR was defined as the proportion of patients who were assessed as CR, PR, or stable disease in CNS lesions. CNS-PFS was defined as the time from the date of the first dose of furmonertinib to the date of documented CNS disease progression or death with any reason, whichever came first. CNS-DoR was defined as the time from the date of first documented CNS-ORR to the date of CNS disease progression or all-cause death, whichever came first.

An exploratory analysis of circulating tumor DNA (ctDNA) was also conducted to reveal more biological information during the study. The analysis included qualitative detection of biomarkers in ctDNA at baseline, plasma *EGFR* T790M mutation clearance rate after six weeks of treatment, and their predictive roles in CNS efficacy of furmonertinib.

### Statistical analysis

The determination of sample size of the two studies could be found in published articles by Shi Y et al. [16, 17]. In this pooled analysis, the CNS-ORR and CNS-DCR were calculated on the basis of the confirmed best response of CNS lesions during the study, and the two-sided confidence intervals (CIs) were performed with Clopper-Person exact method. For time-to-event endpoints (CNS-PFS and CNS-DoR), Kaplan–Meier method was used to determine the median values and corresponding two-sided 95% CIs. Hazard ratios (HRs) of CNS-PFS were calculated using COX proportional hazards model, corresponding *p* values and two-sided 95% CIs were calculated using Wald test. All the statistical analyses were done with SAS (version 9.4, SAS Institute Inc., Cary, NC, USA).

## Results

### Patients

**Fig. 1:**
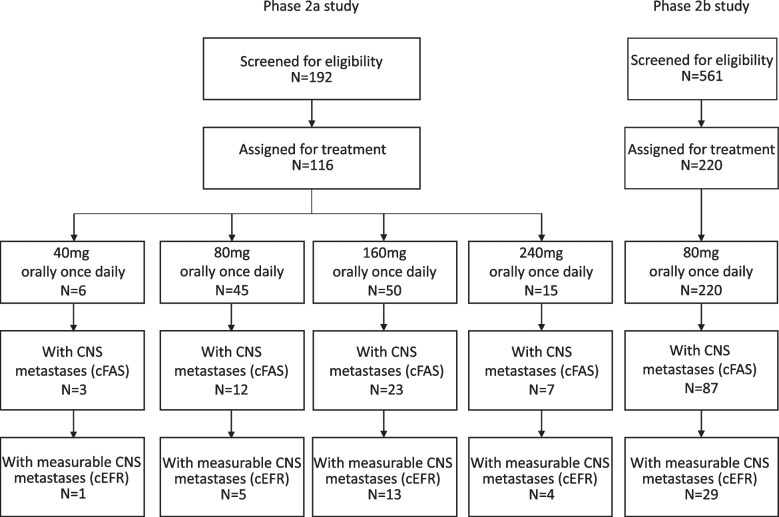
Study flowchart of furmonertinib. CNS, central nervous system. cFAS, CNS full analysis set. cEFR, CNS evaluable-for-response set

A total of 116 patients were enrolled in the phase 2a study from June 1, 2017 to July 24, 2018, of which 45 (39%) patients with CNS metastases at baseline were included in this analysis. Two hundred and twenty patients were enrolled in the phase 2b study from June 4 to December 8 in 2018, and 87 (40%) patients with CNS metastatic at baseline were analyzed in this report. In total, 132 patients with measurable or non-measurable CNS lesions constituted the cFAS and 52 patients with measurable CNS lesions were included in the cEFR population. In the cFAS, three patients received furmonertinib 40 mg orally once daily, 99 patients received furmonertinib 80 mg orally once daily, 23 patients received furmonertinib 160 mg orally once daily, and seven patients received furmonertinib 240 mg orally once daily. In the cEFR, the numbers of patients who were assigned to the groups of furmonertinib 40 mg, 80 mg, 160 mg, and 240 mg orally once daily were one, 34, 13, and four, respectively (Fig. 1). Fifty-five per cent (72/132) of the patients had more than three CNS lesions at baseline. Detailed patient baseline characteristics in the cFAS were shown in Table 1.

**Table 1:** Patients’ baseline characteristics in the cFAS

Characteristics	Groups			
	40 mg orally once daily (*n* = 3)	80 mg orally once daily (*n* = 99)	160 mg orally once daily (*n* = 23)	240 mg orally once daily (*n* = 7)
Age, year, median (range)	47 (44–55)	60 (30–78)	53 (39–67)	55 (37–74)
Sex				
Male, n(%)	1 (33)	41 (41)	9 (39)	2 (29)
Female, n(%)	2 (67)	58 (59)	14 (61)	5 (71)
*EGFR* mutation subtype				
19Del, n(%)	0	54 (55)	12 (52)	4 (57)
L858R, n(%)	3 (100)	42 (42)	11 (48)	3 (43)
19Del + L858R, n(%)	0	1 (1)	0	0
Other, n(%)^a^	0	2 (2)	0	0
Number of CNS lesions				
1–3, n(%)	2 (67)	46 (46)	8 (35)	4 (57)
≥ 4, n(%)	1 (33)	53 (54)	15 (65)	3 (43)
CNS radiotherapy history, n(%)	0	19 (19)	3 (13)	0
CNS surgery history, n(%)	0	1 (1)	0	0

^a^ Two patients in 80 mg orally once daily group harbored de-novo *EGFR* T790M mutation

*cFAS* CNS full analysis set, *EGFR* epidermal growth factor receptor, *CNS* central nervous system

### Efficacy

**Fig. 2:**
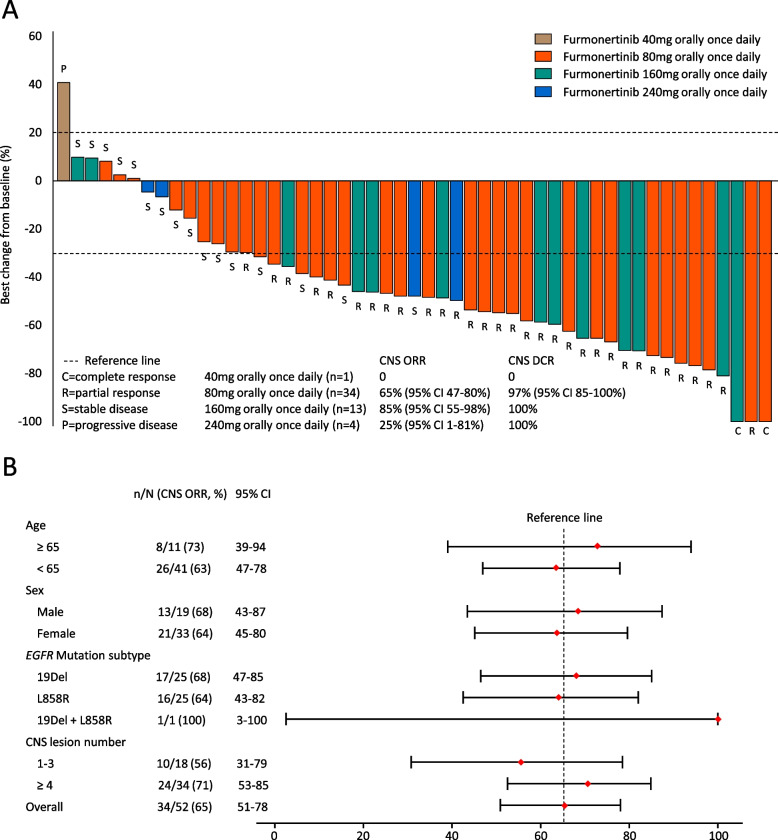
**A** Waterfall plot for the best percentage change of furmonertinib in CNS lesions in the cEFR. The dashed lines at 20% and -30% indicate the thresholds for PD and PR, respectively. One patient in 80 mg orally once daily group was not evaluable. **B** Forest plot of subgroup of patients having objective response of furmonertinib in the cEFR. The reference line at 65% represented the overall CNS-ORR. CNS, central nervous system. cEFR, CNS evaluable-for-response set. PD, progressive disease. PR, partial response. ORR, objective response rate. DCR, disease control rate. CI, confidence interval. EGFR, epidermal growth factor receptor

**Fig. 3:**
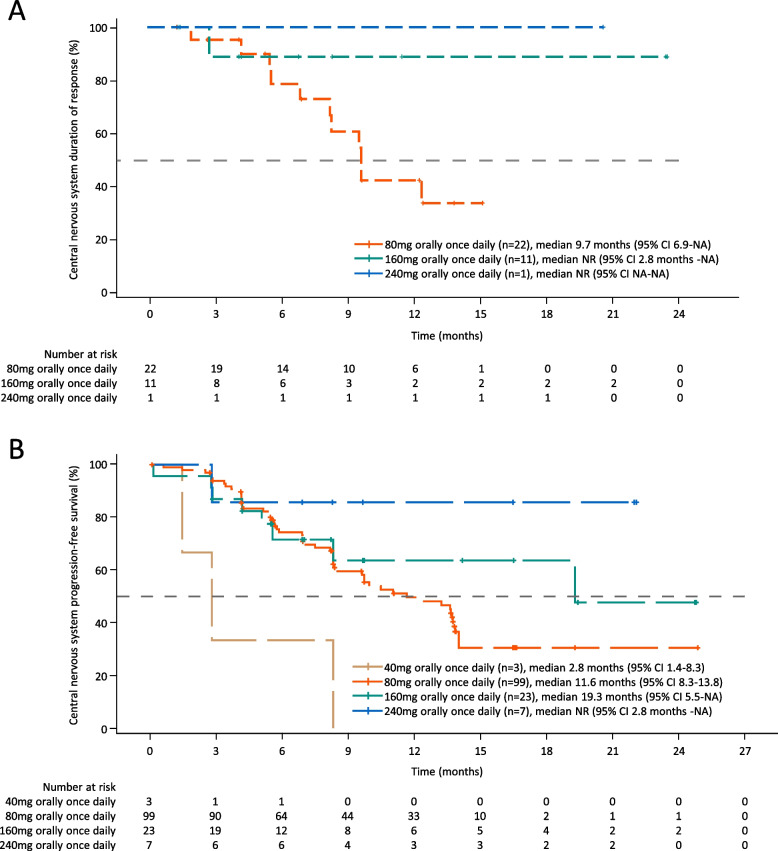
CNS-DoR and CNS-PFS in each group of furmonertinib. **A** CNS-DoR in the cEFR; **B** CNS-PFS in the cFAS. CNS, central nervous system. DoR, duration of response. PFS, progression-free survival. cEFR, CNS evaluable-for-response set. cFAS, CNS full analysis set. NA, not available. NR, not reached. CI, confidence interval

The data cut-off date of this analysis was January 29, 2020. The median follow-up time was 13.5 months (range 0.1–26.1). In the cEFR population, CNS objective response was achieved in zero, 22 (65%), 11 (85%), and one (25%) patient(s) while CNS disease control was documented in zero, 33 (97%), 13 (100%), and four (100%) patient(s) in the 40 mg, 80 mg, 160 mg, and 240 mg orally once daily group, respectively (Table 2, Fig. 2A). The only patient who was assessed as progressive disease (PD) as the best response in CNS lesions was in the lowest dose group (40 mg orally once daily group). The benefit of CNS objective response was consistent in different subgroups (Fig. 2B). In nine patients received brain radiotherapy owing to CNS metastases before enrolment, CNS-ORR was 56% and CNS-DCR was 100%. The median CNS-DoR was 9.7 months (95% CI 6.9-not available [NA]) in the 80 mg orally once daily group and not reached (NR) in the 160 mg and 240 mg orally once daily group, respectively (Fig. 3A). The proportion of patients estimated to be remaining in response at 12 months after the onset of response was 42.6% (95% CI 19.6–64.0%), 88.9% (95% CI 43.3–98.4%), and 100% (95% CI 100–100%) in the 80 mg, 160 mg, and 240 mg orally once daily group, respectively.

**Table 2:** Best CNS response in the cEFR population

CNS response	Groups			
	40 mg orally once daily (*n* = 1)	80 mg orally once daily (*n* = 34)	160 mg orally once daily (*n* = 13)	240 mg orally once daily (*n* = 4)
CR, n (%)	0	1 (3)	1 (8)	0
PR, n (%)	0	21 (62)	10 (77)	1 (25)
SD, n (%)	0	11 (32)	2 (15)	3 (75)
PD, n (%)	1 (100)	0	0	0
NE, n (%)	0	1 (3)	0	0
CNS-ORR (95%CI)	0	65% (47–80%)	85% (55–98%)	25% (1–81%)
CNS-DCR (95%CI)	0	97% (85–100%)	100%	100%

*CNS* central nervous system, *cEFR* CNS evaluable-for-response set, *CR* complete response, *PR* partial response, *SD* stable disease, *PD* progressive disease, *NE* not evaluated, *ORR* objective response rate, *CI* confidence interval, *DCR* disease control rate

In the cFAS population, three (100%), 53 (54%), eight (35%), and one (14%) patient(s) in the 40 mg, 80 mg, 160 mg, and 240 mg orally once daily group had progressed in CNS lesions or died at the data cut-off date, respectively. The corresponding median CNS-PFS was 2.8 months (95% CI 1.4–8.3), 11.6 months (95% CI 8.3–13.8), 19.3 months (95% CI 5.5-NA), and NR (95% CI 2.8 months-NA), respectively (Fig. 3B). CNS-PFS rate at 12 months was 0, 50% (95% CI 38–60%), 64% (95% CI 37–82%), 86% (95% CI 33–98%) in the 40 mg, 80 mg, 160 mg, and 240 mg orally once daily group, respectively.

### Exploratory analysis

**Fig. 4:**
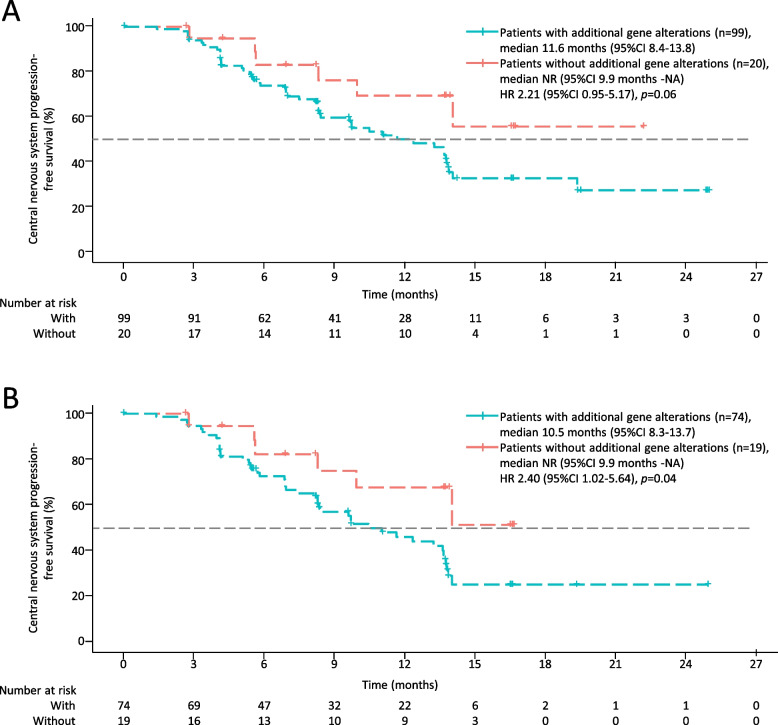
CNS-PFS of patients with and without additional plasma gene alterations at baseline. **A** All doses of furmonertinib; **B** Eighty mg orally once daily of furmonertinib. Plasma gene alterations in addition to *EGFR* mutations including tumor suppressor gene (*PTEN*, *TP53*, or *RB1*) mutations and oncogenic driver gene (*ALK, BRAF, ERBB2*, *KRAS*, *MET*, *RET*, *ROS1, or* amplificated *EGFR*) alterations. CNS, central nervous system. PFS, progression-free survival. CI, confidence interval. NR, not reached. NA, not available. HR, hazard ratio

Of the 132 patients with CNS metastases at baseline enrolled in the phase 2a and 2b studies, 119 patients provided plasma samples for ctDNA testing, and 93 (78%) of these patients were at 80 mg orally once daily dose. Ninety-nine (83%) patients harboured plasma gene alterations in addition to *EGFR* mutations, including six (5%) patients with tumor suppressor gene (*PTEN*, *TP53*, or *RB1*) mutations and 93 (78%) patients with oncogenic driver gene (*ALK, BRAF, ERBB2*, *KRAS*, *MET*, *RET*, or *ROS1*) alterations or amplificated *EGFR* irrespective of tumor suppressor gene mutations. The median CNS-PFS was 11.6 months (95% CI 8.4–13.8) in patients with additional plasma gene alterations and NR (95% CI 9.9 months-NA) in patients who harboured only plasma *EGFR* mutations (HR 2.21 [95% CI 0.95–5.17], *p* = 0.06; Fig. 4A) based on all doses. In the 80 mg orally once daily group (*n* = 93), the median CNS-PFS was 10.5 months (95% CI 8.3–13.7) and NR (95% CI 9.9 months-NA) in patients with and without additional plasma gene alterations, respectively (HR 2.40 [95% CI 1.02–5.64], *p* = 0.04; Fig. 4B).

Among the 119 patients who underwent ctDNA detection at baseline, 84 (71%) patients were plasma *EGFR* T790M mutation positive. Among these 84 patients, their ctDNA was re-evaluated after six weeks of treatment. The plasma *EGFR* T790M mutation was undetectable in 71 (85%) patients at week six (Additional file 1: Figure S1). The clearance rates of plasma *EGFR* T790M mutation were similar between different doses, except for the not-calculatable result in the 40 mg orally once daily group (Additional file 2: Table S1). Among the 84 patients with detectable *EGFR* T790M at baseline, their median CNS-PFS was 13.2 months (*n* = 71, 95% CI 8.4–19.3) in patients with plasma *EGFR* T790M mutation cleared and 10.5 months (*n* = 13, 95% CI 8.4-NA) in patients still harbouring plasma *EGFR* T790M mutation after six weeks of treatment (HR 0.87 [95% CI 0.37–2.08], *p* = 0.77; Additional file 3: Figure S2).

## Discussion

In this post-hoc, pooled analysis of two phase 2 studies, furmonertinib 80–240 mg orally once daily was effective in treating CNS metastatic NSCLC patients with *EGFR* T790M mutation. Numerically better response and longer median CNS-PFS were observed in patients treated by furmonertinib 160 mg orally once daily with limited cases, which warranted further investigation.

CNS metastases represented a risk factor for poor prognosis in *EGFR* mutated NSCLC patients treated with first- or second-generation EGFR TKIs [20]. Third-generation EGFR TKIs were effective in treating *EGFR* T790M mutated NSCLC, but the median PFS and median overall survival (OS) of CNS metastatic patients were still shorter compared with patients without CNS metastases. In a pooled analysis of AURA extension and AURA2 studies, the median PFS treated with osimertinib was 8.2 months (95% CI 6.9–9.7) in CNS metastatic group and 12.4 months (95% CI 9.8–13.8) in CNS non-metastatic group. Similarly, the median OS was 20.3 months (95% CI 17.5–23.6) in CNS metastatic patients and 30.7 months (95% CI 27.5–36.4) in CNS non-metastatic patients [21]. In the phase 3 AURA3 study, the median PFS with osimertinib was 8.5 months (95% CI 6.8–12.3) in CNS metastatic patients and 10.8 months (95% CI 8.3–12.5) in CNS non-metastatic patients [11]. The median OS with osimertinib was 19.2 months (95% CI 16.1–24.3) in patients with CNS metastases and 31.8 months (95% CI 26.6–35.9) in patients without CNS metastases [22]. These results indicated that there were unmet medical needs in *EGFR* T790M mutated, CNS metastatic NSCLC patients.

Several studies explored higher doses or dose escalations of EGFR TKIs to enhance the disease management of CNS metastases. A phase 1 study showed that pulse/continuous-dose erlotinib (1200 mg on days 1–2 and 50 mg on days 3–7 weekly) produced a 75% CNS response rate in *EGFR* mutated lung cancer patients with untreated brain metastases [23]. In the BLOOM study, osimertinib 160 mg orally once daily resulted in a leptomeningeal lesions ORR of 62% and a median PFS of 8.6 months in treated *EGFR* mutated NSCLC with leptomeningeal metastases [24]. In a subcohort analysis with 11 patients from a phase 2 study, the dose escalation of osimertinib to 160 mg orally once daily post intracranial progression on osimertinib 80 mg orally once daily still showed a 54% intracranial response rate and a median intracranial PFS of 8.1 months [25]. Two other reports also indicated the potential of dose escalation of osimertinib in the treatment of CNS progression after the failure on standard dose [26, 27]. Our analysis added to the evidence for the CNS efficacy of higher dose of EGFR TKIs in CNS metastatic, *EGFR* mutated NSCLC patients. However, all of these studies should be interpreted with caution due to the small sample size or the post-hoc or retrospective nature, and larger prospective studies are warranted. Several studies are ongoing to further investigate the efficacy of higher doses of furmonertinib (NCT05465343, NCT05379803) and aumolertinib (NCT04870190) in patients with CNS metastases.

The predictive role of baseline plasma gene alterations in addition of *EGFR* mutations was explored in some studies. The BENEFIT study showed that *EGFR* mutated NSCLC patients with concurrent alterations of tumor-suppressor genes or oncogenes were associated with shorter median PFS when treated with gefitinib [28]. In the phase 2b study of furmonertinib, median PFS was numerically shorter in patients with additional gene alterations besides *EGFR* mutations (including tumor suppressor genes [*TP53, RB1, or PTEN*], oncogenes [*ALK*, *MET, ERBB2, KRAS, BRAF, RET, ROS1, or* amplificated *EGFR*]) than those without at baseline [17]. Similarly, this analysis showed poor predictive effects of accompanying plasma gene alterations on CNS-PFS in patients with *EGFR* T790M mutated NSCLC, indicating that additional gene alterations could also be a potential biomarker in CNS efficacy analysis.

The clearance of plasma *EGFR* T790M mutation after treatment was supposed to predict a better outcome in *EGFR* mutated NSCLC patients. In the osimertinib arm of the AURA3 study, median PFS was significantly longer in patients with plasma *EGFR* mutation clearance at week three than those without (10.9 months [95% CI 8.3–12.7] versus 5.7 months [95% CI 4.1–9.7], HR 0.5 [95% CI 0.3–0.8], *p* = 0.0022) [29]. In the FLAURA study, patients with non-detectable plasma *EGFR* mutations at week three also showed meaningfully longer median PFS than those detected (13.5 months [95% CI 11.1–15.2] versus 9.5 months [95% CI 7.0–10.9], HR 0.57 [95% CI 0.4–0.7], *p* < 0.0001) [30]. However, these studies did not evaluate the relationship between plasma *EGFR* mutation clearance and CNS outcomes. In this analysis, after six weeks of treatment with furmonertinib, plasma *EGFR* T790M clearance was tested and 85% (71/84) patients with plasma *EGFR* T790M clearance did not show a superior CNS-PFS than those without. Additionally, although patients taking higher doses of furmonertinib seemed to have better CNS clinical outcomes, the plasma *EGFR* T790M clearance rate was similar in different groups. These results indicated that plasma *EGFR* T790M clearance might not serve as a predictive biomarker in CNS efficacy of furmonertinib and the possible mechanisms warranted further exploration.

There were some limitations of this report. First, this was a post-hoc analysis which did not exam the statistical difference among different groups, and whether higher doses of furmonertinib could bring better outcomes required further investigation. Second, the efficacy of furmonertinib 240 mg orally once daily did not show a promising ORR possibly due to the small sample size, although the median CNS-PFS was NR with furmonertinib 240 mg orally once daily, and all the patients with 160 mg or 240 mg orally once daily furmonertinib achieved CNS disease control. Third, the definition of CNS-PFS in this pooled post-hos analysis was consistent with previous published CNS analysis of third-generation EGFR TKIs including AURA extension and AURA2 pooled [31], FLAURA [32], FURLONG [19], however, the observed treatment benefit in the CNS-PFS analysis might be influenced as patients did not continue to receive brain scans following disease progression (irrespective of site of progression) or discontinuation from study with no CNS progression or death were censored at date last evaluable CNS assessment.

## Conclusions

In summary, this is an analysis reporting the CNS efficacy of furmonertinib with different doses in *EGFR* T790M mutated NSCLC patients and reveals that furmonertinib of 80 mg orally once daily or higher is effective in CNS metastatic patients. The higher dose of furmonertinib is promising in treating CNS metastases and several studies are ongoing.

## Supplementary Information

**Additional file 1: Figure S1.** VAF of plasma EGFR T790M mutation at baseline and at week six.

**Additional file 2: Table S1.** Plasma EGFR T790M mutation clearance after six weeks of treatment.

**Additional file 3: Figure S2.** Central nervous system progression-free survival of patients with and without plasma EGFR T790M mutation clearance after six weeks of treatment.

## Data Availability

The data underlying this article will be shared on reasonable request to the corresponding author.

## Abbreviations

EGFR: Epidermal growth factor receptor
TKI: Tyrosine kinase inhibitor
CNS: Central nervous system
NSCLC: Non-small cell lung cancer
NA: Not available
PFS: Progression-free survival
FDA: Food and Drug Administration
BM: Brain metastases
ORR: Objective response rate
NGS: Next-generation sequencing
RECIST: Response Evaluation Criteria in Solid Tumors
ECOG: Eastern Cooperative Oncology Group
PS: Performance status
IRC: Independent review center
DCR: Disease control rate
DoR: Duration of response
cFAS: CNS full analysis set
cEFR: CNS evaluable-for-response set
CR: Complete response
PR: Partial response
ctDNA: Circulating tumor DNA
CI: Confidence interval
HR: Hazard ratios
PD: Progressive disease
NR: Not reached
OS: Overall survival
NE: Not evaluated

## References

[1] Shi Y, Au JS, Thongprasert S, Srinivasan S, Tsai CM, Khoa MT (2014). A prospective, molecular epidemiology study of EGFR mutations in Asian patients with advanced non-small-cell lung cancer of adenocarcinoma histology (PIONEER). J Thorac Oncol.

[2] Shi Y, Li J, Zhang S, Wang M, Yang S, Li N (2015). Molecular Epidemiology of EGFR Mutations in Asian Patients with Advanced Non-Small-Cell Lung Cancer of Adenocarcinoma Histology - Mainland China Subset Analysis of the PIONEER study. PloS one..

[3] Maemondo M, Inoue A, Kobayashi K, Sugawara S, Oizumi S, Isobe H (2010). Gefitinib or chemotherapy for non-small-cell lung cancer with mutated EGFR. N Engl J Med.

[4] Zhou C, Wu YL, Chen G, Feng J, Liu XQ, Wang C (2011). Erlotinib versus chemotherapy as first-line treatment for patients with advanced EGFR mutation-positive non-small-cell lung cancer (OPTIMAL, CTONG-0802): a multicentre, open-label, randomised, phase 3 study. Lancet Oncol.

[5] Shi YK, Wang L, Han BH, Li W, Yu P, Liu YP (2017). First-line icotinib versus cisplatin/pemetrexed plus pemetrexed maintenance therapy for patients with advanced EGFR mutation-positive lung adenocarcinoma (CONVINCE): a phase 3, open-label, randomized study. Ann Oncol.

[6] Wu YL, Zhou C, Hu CP, Feng J, Lu S, Huang Y (2014). Afatinib versus cisplatin plus gemcitabine for first-line treatment of Asian patients with advanced non-small-cell lung cancer harbouring EGFR mutations (LUX-Lung 6): an open-label, randomised phase 3 trial. Lancet Oncol.

[7] Wu YL, Cheng Y, Zhou X, Lee KH, Nakagawa K, Niho S (2017). Dacomitinib versus gefitinib as first-line treatment for patients with EGFR-mutation-positive non-small-cell lung cancer (ARCHER 1050): a randomised, open-label, phase 3 trial. Lancet Oncol.

[8] Walter AO, Sjin RT, Haringsma HJ, Ohashi K, Sun J, Lee K (2013). Discovery of a mutant-selective covalent inhibitor of EGFR that overcomes T790M-mediated resistance in NSCLC. Cancer Discov.

[9] Cross DA, Ashton SE, Ghiorghiu S, Eberlein C, Nebhan CA, Spitzler PJ (2014). AZD9291, an irreversible EGFR TKI, overcomes T790M-mediated resistance to EGFR inhibitors in lung cancer. Cancer Discov.

[10] Jänne PA, Yang JC, Kim DW, Planchard D, Ohe Y, Ramalingam SS (2015). AZD9291 in EGFR inhibitor-resistant non-small-cell lung cancer. N Engl J Med.

[11] Mok TS, Wu Y-L, Ahn M-J, Garassino MC, Kim HR, Ramalingam SS (2017). Osimertinib or Platinum-Pemetrexed in EGFR T790M-Positive Lung Cancer. N Engl J Med.

[12] Soria JC, Ohe Y, Vansteenkiste J, Reungwetwattana T, Chewaskulyong B, Lee KH (2018). Osimertinib in Untreated EGFR -Mutated Advanced Non–Small-Cell Lung Cancer. N Engl J Med.

[13] U.S. Food and Drug Administration. AstraZeneca Pharmaceuticals LP, TAGRISSO (osimertinib) [package insert]. https://www.accessdata.fda.gov/drugsatfda_docs/label/2020/208065s021lbl.pdf. Accessed 15 Feb 2023.

[14] Sperduto PW, Kased N, Roberge D, Xu Z, Shanley R, Luo X (2012). Summary report on the graded prognostic assessment: an accurate and facile diagnosis-specific tool to estimate survival for patients with brain metastases. J Clin Oncol.

[15] Fujita Y, Kinoshita M, Ozaki T, Takano K, Kunimasa K, Kimura M (2020). The impact of EGFR mutation status and single brain metastasis on the survival of non-small-cell lung cancer patients with brain metastases. Neurooncol Adv..

[16] Shi Y, Zhang S, Hu X, Feng J, Ma Z, Zhou J (2020). Safety, clinical activity, and pharmacokinetics of alflutinib (AST2818) in patients with advanced NSCLC with EGFR T790M mutation. J Thorac Oncol.

[17] Shi Y, Hu X, Zhang S, Lv D, Wu L, Yu Q (2021). Efficacy, safety, and genetic analysis of furmonertinib (AST2818) in patients with EGFR T790M mutated non-small-cell lung cancer: a phase 2b, multicentre, single-arm, open-label study. Lancet Respir Med.

[18] Shi Y, Chen G, Wang X, Liu Y, Wu L, Hao Y (2022). Furmonertinib (AST2818) versus gefitinib as first-line therapy for Chinese patients with locally advanced or metastatic EGFR mutation-positive non-small-cell lung cancer (FURLONG): a multicentre, double-blind, randomised phase 3 study. Lancet Respir Med.

[19] Shi Y, Chen G, Wang X, Liu Y, Wu L, Hao Y (2022). Central Nervous System Efficacy of Furmonertinib (AST2818) Versus Gefitinib as First-Line Treatment for EGFR-Mutated NSCLC: Results From the FURLONG Study. J Thorac Oncol.

[20] Rangachari D, Yamaguchi N, VanderLaan PA, Folch E, Mahadevan A, Floyd SR (2015). Brain metastases in patients with EGFR-mutated or ALK-rearranged non-small-cell lung cancers. Lung Cancer.

[21] Ahn MJ, Tsai CM, Shepherd FA, Bazhenova L, Sequist LV, Hida T (2019). Osimertinib in patients with T790M mutation-positive, advanced non-small cell lung cancer: Long-term follow-up from a pooled analysis of 2 phase 2 studies. Cancer.

[22] Papadimitrakopoulou VA, Mok TS, Han JY, Ahn MJ, Delmonte A, Ramalingam SS (2020). Osimertinib versus platinum-pemetrexed for patients with EGFR T790M advanced NSCLC and progression on a prior EGFR-tyrosine kinase inhibitor: AURA3 overall survival analysis. Ann Oncol.

[23] Arbour KC, Kris MG, Riely GJ, Ni A, Beal K, Daras M (2018). Twice weekly pulse and daily continuous-dose erlotinib as initial treatment for patients with epidermal growth factor receptor-mutant lung cancers and brain metastases. Cancer.

[24] Yang JCH, Kim SW, Kim DW, Lee JS, Cho BC, Ahn JS (2020). Osimertinib in Patients With Epidermal Growth Factor Receptor Mutation-Positive Non-Small-Cell Lung Cancer and Leptomeningeal Metastases: The BLOOM Study. J Clin Oncol.

[25] Goldstein IM, Roisman LC, Keren-Rosenberg S, Dudnik J, Nechushtan H, Shelef I (2020). Dose escalation of osimertinib for intracranial progression in EGFR mutated non-small-cell lung cancer with brain metastases. Neurooncol Adv..

[26] Piper-Vallillo AJ, Rotow JK, Aredo JV, Shaverdashvili K, Luo J, Carlisle JW (2022). High-Dose Osimertinib for CNS Progression in EGFR+ NSCLC: A Multi-Institutional Experience. JTO Clin Res Rep..

[27] Cordova C, Chi AS, Chachoua A, Kondziolka D, Silverman JS, Shepherd TM (2017). Osimertinib Dose Escalation Induces Regression of Progressive EGFR T790M-Mutant Leptomeningeal Lung Adenocarcinoma. J Thorac Oncol.

[28] Wang Z, Cheng Y, An T, Gao H, Wang K, Zhou Q (2018). Detection of EGFR mutations in plasma circulating tumour DNA as a selection criterion for first-line gefitinib treatment in patients with advanced lung adenocarcinoma (BENEFIT): a phase 2, single-arm, multicentre clinical trial. Lancet Respir Med.

[29] Ahn M, Hartmaier R. Wu Y, Han J, Akamatsu H, John T, et al. FP16.03 early circulating-tumor DNA EGFR mutation clearance in plasma as a predictor of clinical outcomes in The AURA3 Trial. J Thorac Oncol. 2021;16(10):S973–74. 10.1016/j.jtho.2021.08.259

[30] Zhou C, Imamura F, Cheng Y, Okamoto I, Cho BC, Lin MC, et al. Early clearance of plasma EGFR mutations as a predictor of response to osimertinib and comparator EGFR-TKIs in the FLAURA trial. J Clin Oncol. 2019;15_suppl:9020. 10.1200/JCO.2019.37.15_suppl.9020

[31] Goss G, Tsai CM, Shepherd FA, Ahn MJ, Bazhenova L, Crinò L (2018). CNS response to osimertinib in patients with T790M-positive advanced NSCLC: pooled data from two phase II trials. Ann Oncol.

[32] Reungwetwattana T, Nakagawa K, Cho BC, Cobo M, Cho EK, Bertolini A (2018). CNS Response to Osimertinib Versus Standard Epidermal Growth Factor Receptor Tyrosine Kinase Inhibitors in Patients With Untreated EGFR -Mutated Advanced Non–Small-Cell Lung Cancer. JCO.

