# Supplementary material for: Central nervous system efficacy of furmonertinib (AST2818) in patients with EGFR T790M mutated non-small cell lung cancer: a pooled analysis from two phase 2 studies
Source: BMC Med. 2023 Apr 28;21:164. doi: 10.1186/s12916-023-02865-z (PMC10148399; doi:10.1186/s12916-023-02865-z)

Figure S1. VAF of plasma *EGFR* T790M mutation at baseline and at week six.

VAF, variant allele frequency. EGFR, epidermal growth factor receptor

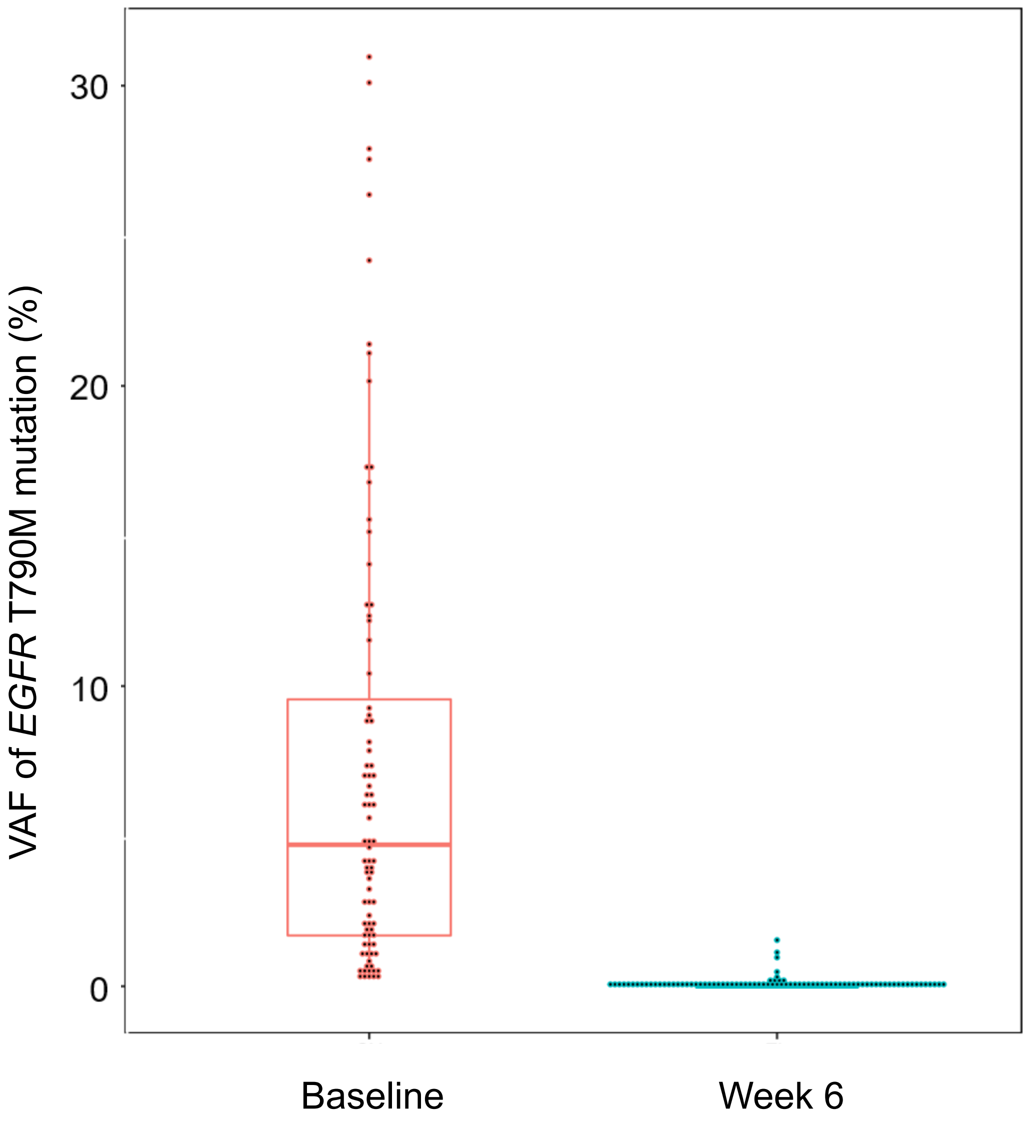

Supplement: Supplementary file 1 — Additional file 1: Figure S1. VAF of plasma EGFR T790M mutation at baseline and at week six. [file 12916_2023_2865_MOESM1_ESM.pdf]
