# Supplementary material for: Central nervous system efficacy of furmonertinib (AST2818) in patients with EGFR T790M mutated non-small cell lung cancer: a pooled analysis from two phase 2 studies
Source: BMC Med. 2023 Apr 28;21:164. doi: 10.1186/s12916-023-02865-z (PMC10148399; doi:10.1186/s12916-023-02865-z)
Supplement: Supplementary file 2 — Additional file 2: Table S1. Plasma EGFR T790M mutation clearance after six weeks of treatment. [file 12916_2023_2865_MOESM2_ESM.docx]

Table S1. Plasma *EGFR* T790M mutation clearance after six weeks of treatment.

| Statistical items | Groups | | | | |
| --- | --- | --- | --- | --- | --- |
|  | 40 mg orally once daily | 80 mg orally once daily | 160 mg orally once daily | 240 mg orally once daily | Total |
| Patients detected for ctDNA at baseline | 0 | 93 | 20 | 6 | 119 |
| Patients with baseline plasma *EGFR* T790M mutation positive and received ctDNA testing after six weeks of treatment | 0 | 61 | 17 | 6 | 84 |
| Patients with plasma *EGFR* T790M mutation clearance after six weeks of treatment | 0 | 52 | 14 | 5 | 71 |
| Plasma *EGFR* T790M mutation clearance rate | Not calculable | 85% | 82% | 83% | 85% |

*EGFR* epidermal growth factor receptor, *ctDNA* circulating tumor DNA
