# Supplementary material for: Central nervous system efficacy of furmonertinib (AST2818) in patients with EGFR T790M mutated non-small cell lung cancer: a pooled analysis from two phase 2 studies
Source: BMC Med. 2023 Apr 28;21:164. doi: 10.1186/s12916-023-02865-z (PMC10148399; doi:10.1186/s12916-023-02865-z)

Figure S2. Central nervous system progression-free survival of patients with and without plasma *EGFR* T790M mutation clearance after six weeks of treatment. EGFR, epidermal growth factor receptor. CI, confidence interval. NA, not available. HR, hazard ratio

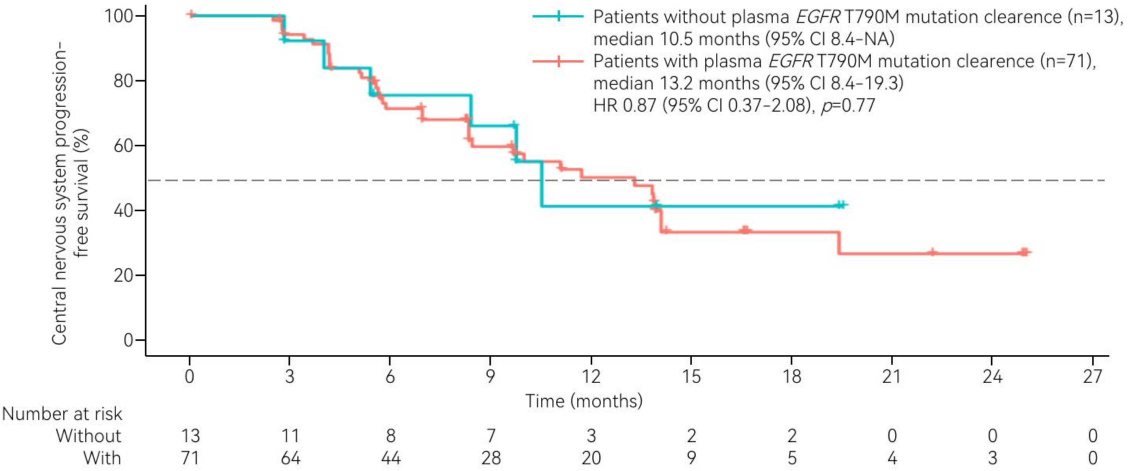

Supplement: Supplementary file 3 — Additional file 3: Figure S2. Central nervous system progression-free survival of patients with and without plasma EGFR T790M mutation clearance after six weeks of treatment. [file 12916_2023_2865_MOESM3_ESM.pdf]
